# Peri‐operative identification and management of patients with unhealthy alcohol intake

**DOI:** 10.1111/anae.16530

**Published:** 2025-01-09

**Authors:** Matthew J. A. Jenkins, Stephen M. Kinsella, Matthew D. Wiles, Brijesh Srivastava, Catherine Griffiths, Jacquelyn Lewin, Stephen Usher, Gautam Mehta, Abi Berger, Dereck Gondongwe, Isra Hassan

**Affiliations:** ^1^ Consultant, Department of Anaesthesia, Pain and Peri‐operative Medicine Te Whatu Ora Counties Manukau Health Auckland New Zealand; ^2^ Consultant, Department of Anaesthesia University Hospitals Bristol and Weston NHS Foundation Trust Bristol UK and Co‐Chair representing the Association of Anaesthetists; ^3^ Consultant, Department of Anaesthesia and Operating Services Sheffield Teaching Hospitals NHS Foundation Trust UK; ^4^ Fellow, Centre for Applied Health and Social Care Research Sheffield Hallam University Sheffield UK; ^5^ Consultant Hepatologist, Cardiff and Vale NHS Trust Cardiff UK; ^6^ Resident Doctor, Department of Anaesthesia Aneurin Bevan University Health Board Newport UK; ^7^ Consultant, Department of Anaesthesia New Cross Hospital, Royal Wolverhampton Hospital NHS Trust Wolverhampton UK; ^8^ Consultant, Department of Anaesthesia Cardiff and Vale NHS Trust Cardiff UK; ^9^ Associate Professor, Institute for Liver and Digestive Health University College London London UK; ^10^ Honorary Consultant Hepatologist, Royal Free London NHS Foundation Trust London UK; ^11^ General Practitioner, NHS Fitzrovia Medical Centre London UK; ^12^ Deputy Lead Pharmacist, Critical Care Division University College London Hospitals NHS Trust London UK; ^13^ Consultant, Department of Peri‐operative Medicine, University College London Hospitals NHS Trust London UK; ^14^ Consultant, Department of Anaesthesia, Cardiff and Vale NHS Trust Cardiff UK and Co‐Chair of the Working Party

**Keywords:** alcohol, alcohol‐related disorders, anaesthesia, liver disease, surgery

## Abstract

**Introduction:**

This consensus statement gives practical advice for the safe management of patients with harmful alcohol intake undergoing elective and emergency surgery. The wide spectrum of alcohol‐related organ dysfunction observed in this cohort of patients may have a profound impact on care, and the additional effects of alcohol withdrawal may further exacerbate postoperative morbidity and mortality.

**Methods:**

A working party was assembled based on clinical and/or academic expertise in the area. Recommendations were formulated using a modified Delphi process. An initial list of recommendations was produced following targeted literature reviews for all relevant phases of patient care throughout the peri‐operative pathway. These recommendations were distributed among the authors who rated each as ‘include’, ‘exclude’; or ‘revise’. Recommendations with ≥ 75% inclusion decision were included.

**Results:**

The working party produced a list of 10 key peri‐operative management recommendations. These include recommendations on how to screen effectively for excessive alcohol usage in the surgical population. To achieve this, a validated point‐of‐care tool is used with additional weighting provided by considering surgical urgency. This is combined with the use of scoring systems to facilitate decisions regarding peri‐operative care including postoperative location. This document also provides clear explanation of the physiological and pharmacological issues relating to alcohol excess, highlighting the direct effects of alcohol and its secondary effects on organ systems.

**Discussion:**

This consensus statement offers strategies and solutions to minimise the impact of harmful alcohol intake on the safe conduct of anaesthesia.

## What other guideline statements are available on this topic?

There are no other nationally or internationally agreed guidelines for screening, identification and standardised management of patients with excessive alcohol consumption who are having elective and emergency surgical procedures. Accepting that the focus of this consensus statement is on the peri‐operative management of patients with excessive alcohol consumption, we have also drawn on evidence related to hepatic disease and its anaesthetic implications.

## Why were these guidelines developed?

Patients with unhealthy alcohol intake represent a high‐risk surgical cohort, having an increased probability of peri‐operative complications, mortality and long‐term functional decline following surgical procedures. Many of the problems in this group of patients can be minimised by timely identification and pre‐emptive management, thereby improving outcomes and potentially reducing healthcare expenditure.

## How and why does this statement differ from other national guidelines?

In developing this document, we have incorporated elements from national guidelines relating to non‐alcoholic liver disease and alcohol issues in the non‐surgical population. These resources are primarily from the National Institute for Health and Care Excellence (NICE) that describe relevant interventions but do not frame these within a peri‐operative context.

## Recommendations

- Patients should be screened during pre‐assessment for surgery using an appropriate tool such as the alcohol use disorders identification test consumption (AUDIT) questionnaire to triage risk relating to alcohol use.
- Those patients with harmful chronic alcohol intake or who are judged to be at high risk of alcohol‐related liver disease should have careful pre‐operative physical examination and additional relevant investigations. Harmful intake is suggested by an AUDIT score > 19 and/or consumption of > 35 units of alcohol per week (women) or > 50 units of alcohol per week (men).
- In pre‐operative patient populations, elevated AUDIT questionnaire scores should prompt a proportional response ranging from brief intervention to inpatient specialist referral.
- It should be borne in mind that patients who have previously demonstrated harmful alcohol consumption may still be at high risk of alcohol‐related liver disease irrespective of current screening scores.
- Risk assessment and decision‐making in established alcohol‐related liver disease should be guided via the use of validated scoring systems such as the Surgical Outcome Risk Tool (SORT).
- The peri‐operative clinician managing harmful alcohol intake should consider surgical urgency according to recognised classification systems. It may be appropriate to delay some surgical procedures to allow interventions that reduce the risk of alcohol‐related complications. Even emergency surgical populations may benefit from screening for alcohol‐related risk and commensurate intervention to mitigate this.
- Patients with chronic harmful alcohol intake may be appropriate for day‐case surgery. Potential barriers to suitability are organ dysfunction, social circumstances and concerns relating to immediate recommencement of alcohol consumption following day‐case discharge. These should be carefully investigated and discussed.
- Analgesic techniques that reduce the requirements for opioids, including regional methods if appropriate, should be considered. Patients with cirrhosis and alcoholic hepatitis are at elevated risk of complications when opioids are used. Specialist assistance from acute pain services should be considered, while the prescription of other commonly used analgesics may need to be modified or avoided altogether in these cases.
- Patients with harmful alcohol intake are at high risk of postoperative complications such as infections, arrhythmias, bleeding and delirium. The development of these complications should be actively monitored with due consideration for critical care admission.
- At‐risk patients should undergo regular peri‐operative assessment for alcohol withdrawal. Interventions should be guided by objective scoring systems, such as the Clinical Institute Withdrawal Assessment Alcohol—Revised (CIWA‐Ar) scale, within a defined care pathway. Patients with underlying liver cirrhosis and/or who are older are at risk of benzodiazepine‐related complications; short‐acting drugs should be employed to manage withdrawal in these cases.

## Introduction

Elevated or harmful alcohol consumption is common in England. In 2019, 30% of men and 15% of women were documented as regularly drinking more than the recommended safe weekly limits for alcohol consumption [1]. Such data are proportionally reflected in the 2% of all 2019–2020 NHS admissions for which alcohol was a primary reason for inpatient stay [1].

**Figure 1:**
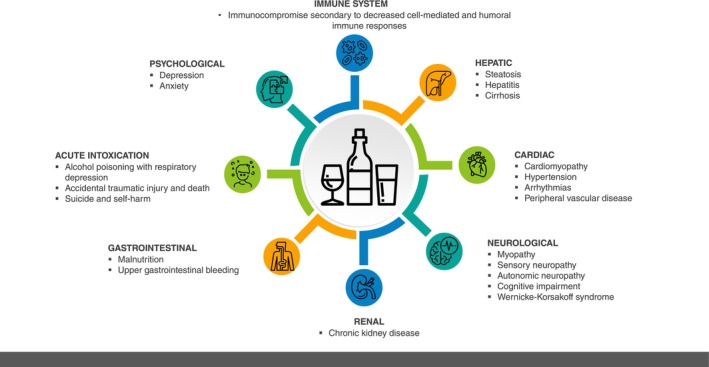
Systemic complications of unhealthy alcohol intake.

**Figure 2:**
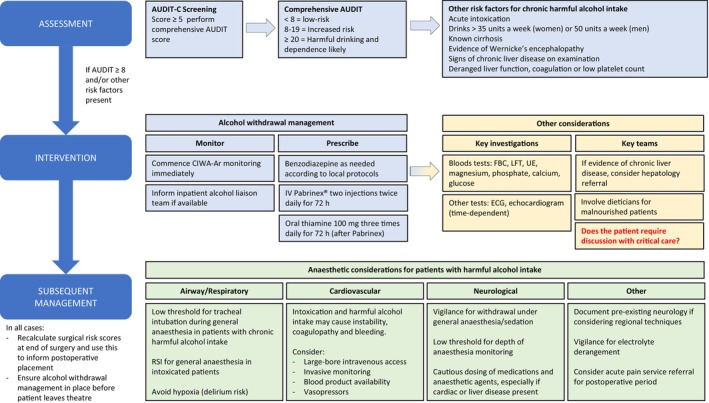
Summary of peri‐operative alcohol management guidelines for adult patients undergoing emergency surgery. AUDIT‐C, alcohol use disorders identification test consumption; AUDIT, alcohol use disorders identification test; CIWA‐Ar, Clinical Institute Withdrawal Assessment for Alcohol – revised; FBC, full blood count; UE, urea and electrolytes; LFT, liver function tests; ECG, electrocardiogram; RSI, rapid sequence induction.

**Figure 3:**
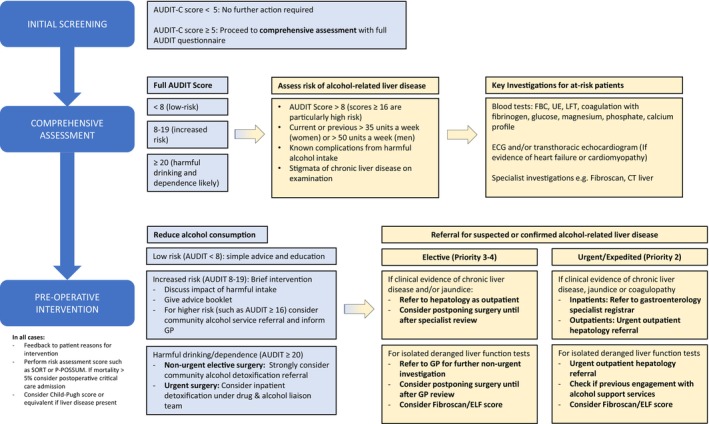
Summary of peri‐operative alcohol management guidelines for adult patients undergoing elective surgery. AUDIT‐C, alcohol use disorders identification test consumption; AUDIT, alcohol use disorders identification test; FBC, full blood count; UE, urea and electrolytes; LFT, liver function tests; ECG, electrocardiogram; CT, computed tomography; SORT, surgical outcome risk score; P‐POSSUM, Portsmouth physiological and operative severity Score for the enumeration of mortality and morbidity; GP, general practitioner; ELF, enhanced liver fibrosis.

Chronic harmful alcohol intake causes a spectrum of organ dysfunction of which alcohol‐related liver disease (including cirrhosis) remains the most common cause of death [2]. This heterogeneity reflects the myriad systemic effects that may complicate management from both chronic alcohol intake and acute intoxication respectively, with a range of clinical implications (Fig. 1) [3]. These issues justify proactive identification and treatment of such patients, who may have clinically occult, yet potentially impactful, liver disease. A structured, multidisciplinary approach is appropriate throughout the peri‐operative period, with due modification according to surgical priority (Figs. 2 and 3) [4].

## Methods

We aimed to produce a multidisciplinary consensus statement directed by a working party with a diverse authorship who were invited based on their clinical and/or academic expertise in the area. Recommendations were formulated using a modified Delphi process. An initial list of recommendations was produced following targeted literature reviews for all relevant phases of patient care throughout the peri‐operative pathway. These recommendations were distributed among the authors who rated each as ‘include’, ‘exclude’ or ‘revise’, as well as providing anonymised comments onto a Microsoft Excel spreadsheet (Microsoft Inc., Redmond, WA, USA). Recommendations with ≥ 75% inclusion decision were included.

## Results

### Assessment

**Figure 4:**
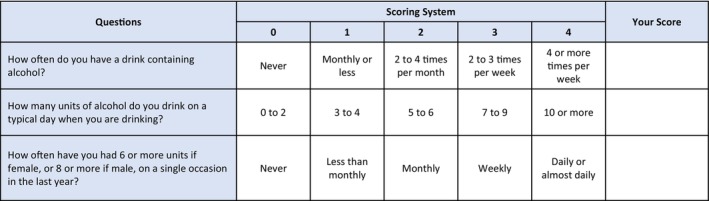
Alcohol use disorders identification – consumption (AUDIT‐C).

**Figure 5:**
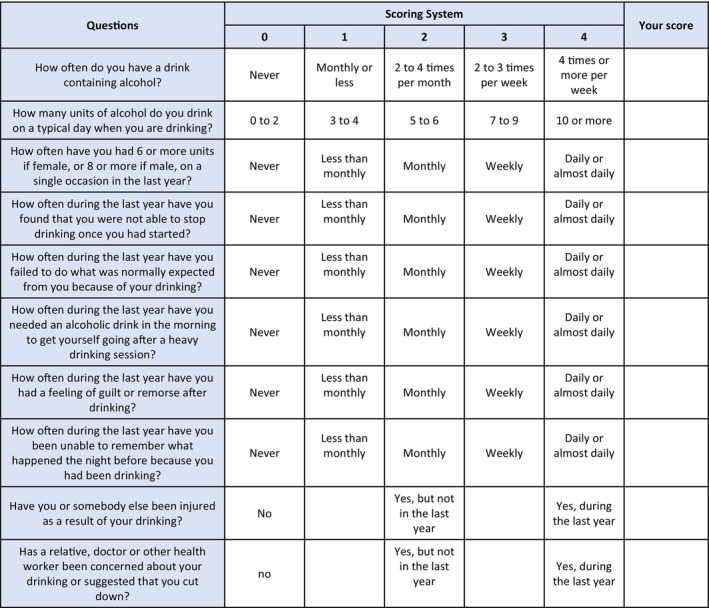
Alcohol use disorders identification test (AUDIT).

Current literature recommends that all patients are screened for alcohol intake as part of routine practice [5, 6]. In the peri‐operative setting, this aligns with a recent target from the Commissioning for Innovation and Quality (CQUIN) framework advising that 80% of patients with a hospital stay of one night should be screened for alcohol use using a validated screening tool [7]. Of the available tools, the abbreviated alcohol use disorders identification test – consumption (AUDIT‐C) (Fig. 4) combines high sensitivity and specificity with rapid use [8, 9]. It comprises three self‐reported questions highlighting at‐risk patients (score ≥ 5), who would benefit from comprehensive assessment of alcohol consumption by a healthcare professional using seven further questions from the full abbreviated alcohol use disorders identification test (AUDIT) questionnaire (Fig. 5) [6, 9].

Patients who score < 5 on the abbreviated questionnaire or < 8 on comprehensive assessment, are deemed at lower risk [5] and can continue on the normal patient pathway. Patients who score 8–19 on comprehensive AUDIT assessment are at increased risk of alcohol‐related liver disease. In addition, a score of > 19 is consistent with harmful drinking and may indicate alcohol dependence. This is also suggested by consumption of > 35 units of alcohol per week (female sex) or > 50 units of alcohol per week (male sex). If there is a significant history of harmful drinking in the past in terms of units consumed per week, then patients may potentially still be high risk regardless of the current score on the questionnaire.

Patients with alcohol‐related liver disease should be evaluated using established scoring systems to guide peri‐operative risk quantification and shared decision‐making. Externally validated risk calculators such as P‐POSSUM and SORT may be corroborated with disease‐specific systems such the Child‐Turcotte‐Pugh score or surgical risk metrics such as the Mayo Risk Score or the VOCAL‐Penn score [10, 11, 12]. The VOCAL‐Penn score has been externally validated in patients with cirrhosis and found to have superior discrimination compared with other related tools [13]; it can be calculated online at http://www.vocalpennscore.com.

### Physical examination

In addition to a standard assessment, at‐risk patients should be examined for stigmata of chronic liver disease. In particular, assessment for ascites or asterixis (as a sign of hepatic encephalopathy) should be undertaken as these represent decompensation of cirrhosis which may require specific management. Hypertension is common with alcohol excess, although patients with cirrhosis may have low blood pressure due to hyperdynamic circulation and reduced systemic vascular resistance. It should be noted that findings in liver disease may range from normal to overt decompensation depending on the severity of the disease [8]. When considering regional techniques, it is prudent to document pre‐existing weakness or neurological abnormalities as this may be relevant in the assessment of post‐procedural complications such as nerve injury.

### Investigations

A full blood count is useful since anaemia (defined in the peri‐operative setting as haemoglobin < 130 g.l^‐1^ [14]) from a variety of causes is common in harmful drinking. Although this does not invariably represent a haematinic issue, macrocytic anaemia with a raised mean corpuscular volume may indicate folate/vitamin B12 deficiency or bone marrow toxicity [8]. Iron deficiency may predominate, with reduced ferritin or transferrin/iron saturation levels; however, it should be remembered that ferritin is often artefactually elevated in patients with raised alcohol consumption. Reciprocally, the absence of microcytosis does not mean the patient will not be iron deficient as peripheral erythrocytes may exist in various stages of development. The red cell distribution width may be helpful in these cases as a diagnostic adjunct.

Thrombocytopaenia may be present secondary to bone marrow suppression, folate deficiency or hypersplenism, possibly reflecting underlying portal hypertension. Leucocytosis may also be present due to hepatitis‐related leukaemoid reaction [15]. As discussed, severity of liver disease may be assessed by the Child‐Turcotte‐Pugh score. Key components of this are serum albumin; serum bilirubin; and prothrombin time. Additionally, elevated (conjugated) bilirubin may suggest active alcoholic steatohepatitis, even in the absence of cirrhosis.

Electrolyte disturbances, particularly hyponatraemia, are common with alcohol excess even in the absence of advanced or cirrhotic liver disease. Hypokalaemia and hypophosphataemia may cause muscle weakness, while hypomagnesaemia may worsen hypokalaemia and may cause seizures [15]. Urea and creatinine can be elevated by pre‐renal causes of acute kidney injury more commonly seen in patients with cirrhosis, while hepatorenal syndrome only occurs in the setting of ascites or significant portal hypertension. Isolated uraemia may be due to active gastrointestinal bleeding [15].

Biochemical markers for alcohol‐related liver disease have been employed historically to assess liver dysfunction; however, abnormal liver enzymes are poorly predictive of underlying liver disease with only 3.9% of patients with an abnormal value in this domain shown to develop significant liver disease within 5 years of testing [6, 15, 16]. Liver enzyme values should, therefore, be interpreted in the context of wider clinical findings and other test results.

Harmful alcohol intake increases the risk of cardiovascular disease ranging from hypertension and atrial arrhythmias to more serious problems such as cardiomyopathy. The incidence of stroke (haemorrhagic or ischaemic) is also increased [17]. Diagnosis can be aided by using a 12‐lead ECG, while echocardiography can show structural damage to the heart caused by alcohol misuse such as a dilated left ventricle with decreased mass and wall thickness or systolic dysfunction. Echocardiography should be reserved for those patients with recognised criteria such as those displaying signs and symptoms of cardiac failure [18]. In selected cases, cardiopulmonary exercise testing may be of value to identify systolic dysfunction that is masked by the hyperdynamic circulation of liver impairment [19].

Other more technical investigations to identify a fatty or cirrhotic liver such as ultrasound, computed tomography or liver biopsy are best organised by a gastroenterologist after appropriate referral [8]. The use of transient elastography to measure liver stiffness (e.g. Fibroscan®, Echosens, France) in patients who are alcohol‐dependent has gained particular diagnostic prominence as reflected by recent commissioning data and national guidelines [20, 21]. Depending on local availability, some institutions may employ alternatives to elastographic testing, such as the enhanced liver fibrosis test [22], in the assessment of hepatic fibrosis risk. Establishing the presence of underlying advanced fibrosis/cirrhosis is important due to its impact on relevant peri‐operative outcomes and the risk of hepatic decompensation. Additionally, transient elastography is now used to stratify the risk of complications from portal hypertension; in this context liver stiffness < 20 kPa and platelet count > 150 × 10^9^.l^‐1^ are accepted thresholds to rule out the presence of large oesophageal varices in compensated cirrhosis [23].

### Harmful alcohol use and older patients

Age‐related changes in organ function, combined with comorbidity and depleted physiologic reserve, render older patients particularly vulnerable to the effects of chronic excessive alcohol use and some related management interventions such as long‐acting benzodiazepines. This is compounded by the increasing frequency and volume of alcohol consumption observed in populations aged 65–74 y. Harmful alcohol intake is an independent risk factor for the development of frailty and is strongly associated with dementia, falls and delirium [24]. Additional risk factors for harmful chronic alcohol intake may be observed in patients > 65 y including: male sex; single marital status; social isolation; insomnia; depression; dementia; chronic pain; and substance availability [24].

### Intervention

#### In the community

In elective settings, safe pre‐operative reduction in alcohol intake remains a core element of management. The level of intervention to achieve this is influenced by the degree of patient risk as determined by objective scoring or weekly alcohol intake.

Pre‐operative assessment represents an ideal opportunity to make a brief intervention (a ‘teachable moment’) among those patients scoring 8–19 on the AUDIT score to discuss the potential harm from excessive alcohol intake and provide advice leaflets. Increased risk scores should prompt a recommendation that patients self‐refer to community alcohol support services [5, 25, 26]. Clinicians should have lower intervention thresholds for patients with additional risk factors such as female sex, patients aged < 18 or > 65 y, and those from Black and minority ethnic backgrounds [5].

Patients scoring > 19 on comprehensive AUDIT have possible alcohol dependency [5, 25] and should be referred for community‐based assisted withdrawal [27]. This requires close liaison with local alcohol support services and primary care to ensure that it is carried out in a timely manner before admission for surgery.

Those individuals who are judged to be vulnerable or are aged ≤ 16 y may need to be referred as an inpatient for specialist support [27] and possible inpatient alcohol withdrawal by hospital alcohol support services (or gastroenterology teams where these are not available). A lower threshold for controlled in‐hospital detoxification may also be applied for cases where community management is superseded by operative urgency.

Local service availability, expertise and referral pathways may vary such that a community withdrawal programme may be preferred rather than inpatient admission in some institutions. Ownership from named, contactable clinicians or services from the outset is important to promote continuity of care in all settings. Parent teams should review patient progress regularly and intervene as needed to ensure effective, safe management. Finally, the refusal of a competent patient to engage in discussed interventions should not be treated as an absolute contraindication to surgery provided there has been an appropriate discussion about related risks on a case‐by‐case basis.

#### Inpatients

Inpatient specialist referral may be warranted for patients admitted with harmful drinking or alcohol‐dependence who are experiencing acute withdrawal or at high risk of this (Box 1) [27, 28]. This includes urgent/emergency cases and patients admitted for planned detoxification. Close liaison aids early awareness of any potential issues but also facilitates assessment of community withdrawal success and guides the decision for further support.

Box 1 **Clinical features of acute alcohol withdrawal syndrome. Adapted from [24]**

**Alcohol withdrawal syndrome**

- Acute alcohol withdrawal syndrome describes a spectrum of clinical sequelae typically occurring after abrupt cessation of alcohol consumption in patients with chronic, harmful intake.
- It has been attributed to impaired neurotransmitter regulation in the central nervous system, whereby chronically elevated cerebral ethanol concentrations decrease sensitivity to inhibitory gamma‐amino butyric acid (GABA) and interfere with glutamate receptor binding, causing compensatory upregulation of excitatory N‐methyl‐D‐Aspartate (NMDA) receptors. The sudden absence of ethanol in this context results in a pro‐excitatory imbalance that causes many of the observed symptoms and signs during withdrawal states.
- Minor symptoms typically occur within 6 h and include insomnia; tremor; anxiety; gastrointestinal upset; headache; diaphoresis; and palpitations. More severe features, described below, may occur in some patients.

**Withdrawal seizures and delirium tremens**

- Generalised tonic–clonic seizures may occur, most commonly in patients aged 40–50 years with prolonged harmful intake. Seizure activity is most common 12–48 h following cessation of alcohol intake.
- If untreated, one third of alcoholic seizures may progress to delirium tremens, characterised by hypertension; tachycardia; confusion; hallucinations; agitation; and diaphoresis. It is distinct from isolated alcoholic hallucinations, which are not usually accompanied by confusion or deranged vital signs.
- With appropriate recognition and management, the mortality from delirium tremens is now < 5%.

**Metabolic abnormalities**

- Patients may be hypovolaemic and demonstrate a metabolic acidosis, frequently accompanied by electrolyte abnormalities such as hypokalaemia; hypophosphataemia; and hypomagnesaemia.
- Deranged metabolic findings in this group are often multifactorial and interrelated, and themselves may result in organ dysfunction and an increased risk of cardiac failure, arrhythmias, reduced seizure threshold and mortality.

**Figure 6:**
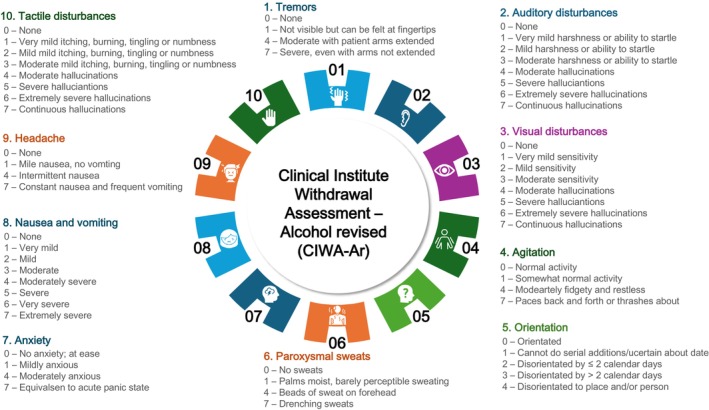
Components of the Clinical Institute Withdrawal Assessment Alcohol – Revised (CIWA‐Ar) Scale.

On admission to hospital, the revised Clinical Institute Withdrawal Assessment – Alcohol (CIWA‐Ar) score should be measured in at‐risk patients [25, 27]. This is a 10‐point scale (Fig. 6) used to assess alcohol withdrawal that can be used to direct pharmacological interventions; benzodiazepines such as chlordiazepoxide are a mainstay of therapy, while alternatives include carbamazepine and clomethiazole [27, 29]. The required interval between medication doses will vary but is often every 90 min with administration of a longer‐acting benzodiazepine, such as diazepam 20 mg, triggered at a CIWA‐Ar score of > 11 [29]. This is repeated until there are three consecutive CIWA‐Ar scores < 11, indicating complete detoxification. The patient should then continue with standard vital and neurological observations unless there is a re‐emergence of symptoms. This strategy may warrant modification in established liver cirrhosis due to the concurrent risk of encephalopathy; in this setting the exclusive use of short‐acting benzodiazepines such as oxazepam is warranted. This may also apply to other groups who can have greater sensitivity to sedative drugs, such as patients who are older.

Seizures are a common sequel of harmful alcohol intake, particularly in the context of withdrawal. Other differentials, such as hypoglycaemia or electrolyte deficiencies, should be considered as part of management. Alcohol‐related seizures occurring as an inpatient should be dealt with according to established guidelines [30]. Benzodiazepines are a mainstay of therapy; phenytoin should be avoided [27]. Following a seizure the patient should continue, or recommence, CIWA‐Ar scoring as appropriate.

### Type of surgery

#### Day‐case surgery

There are few published data relating to day surgery in patients with harmful alcohol intake. However, day‐case procedures are potentially advantageous in this group as they can avoid the withdrawal states associated with inpatient hospital stays. Many general aspects of management are encompassed within pre‐existing guidelines [31]. At all stages, appropriate involvement of the multidisciplinary team is vital for decision‐making regarding day‐case suitability and timing of other elements, such as controlled withdrawal programmes. Case‐by‐case decision making may vary depending on individual patient characteristics, local resources and personnel, particularly when planning safe postoperative discharge.

In high‐volume, nurse‐led pre‐assessment settings, the addition of the AUDIT‐C to other commonly used scoring systems provides a valuable, rapidly applicable measure for seeking further clinician involvement such as discussion with an anaesthetist. Day surgery‐specific pre‐operative assessment should otherwise be undertaken by practitioners competent in eliciting symptoms and signs indicative of complications from chronic alcohol use. Cardiovascular and hepatic manifestations such as dilated cardiomyopathy and cirrhosis are particularly relevant to day‐case suitability. The presence of coagulopathy or thrombocytopenia, with their attendant bleeding risks, should also influence decision‐making. It may be safer for some patients to be managed as inpatients, depending on the nature of the planned surgical intervention and outcomes of appropriate multidisciplinary discussions.

As for inpatient surgery, consideration should be given to deferring surgery for planned community withdrawal under appropriate supervision for all high‐risk patients. Potential benefits from abstinence for at least 2 weeks include improved platelet function and therefore reduced bleeding risk, while cessation of alcohol intake for 8 weeks or longer may improve wound healing [32]. Patients may refuse to engage with a pre‐operative intervention as well as advice for the postoperative period, such as non‐consumption of alcohol for 24 h. This may affect the patients' appropriateness for day‐case procedures and should carefully be explored, with appropriate risk/benefit discussions and development of an individualised management plan in consultation with the wider team. Patients who present acutely intoxicated on the day of surgery should have surgery postponed with appropriate follow up.

There may be a particular role for regional techniques, including peripheral nerve blockade if feasible and safe, to facilitate same‐day discharge while potentially reducing home analgesic requirements. The use of short‐acting anaesthetic drugs provides an additional margin of safety by minimising the risk of pharmacological interaction with alcohol consumed after surgery. Caution should be exercised when prescribing take‐home postoperative systemic drugs where there is evidence of alcohol‐related liver disease (Table 1).

**Table 1:** Peri‐operative considerations for systemic analgesia in patients with harmful alcohol intake with or without established alcoholic liver disease. Adapted from [33, 34].

Drug	Issue	Action
Paracetamol	Depletion of glutathione stores in established liver disease increases vulnerability to NAPQI‐toxicity	Avoid paracetamol administration altogether in patients with advanced alcohol‐related liver disease who still consume alcohol
	Continued harmful alcohol intake results in P450 enzyme induction and increased NAPQI generation	Limit paracetamol dose to 2 g per 24 h in patients with cirrhosis who no longer consume alcohol, or patients without cirrhosis who have chronic harmful alcohol intake
Non‐steroidal anti‐inflammatory drugs	Administration increases risk of gastrointestinal bleeding, variceal haemorrhage, impaired renal function and development of drug‐resistant ascites	Avoid in established alcohol‐related liver disease
Opioids	Bioavailability of orally administered opioids is increased due to reduced first‐pass metabolism	Initial dose and frequency of morphine prescription should be reduced by 50% and titrated upwards to effect
	Reduced metabolism risks accumulation, especially that of opioids with active metabolites such as morphine, where half‐life may be increased by up to two‐fold	Tramadol dose should be reduced to 25 mg every 8 h in patients with established alcohol‐related liver disease. It should be avoided completely in patients who are at risk of seizures, such as those experiencing withdrawal
	Tramadol also has active metabolites which may accumulate and may lower the seizure threshold in at‐risk patients	Fentanyl is a generally the best choice due to its short action and inactive metabolites. Single doses do not require modification, but repeated doses, increased frequency or infusions still have potential to accumulate
		Codeine should not be administered due to unpredictable prodrug metabolism

NAPQI, N‐acetyl‐p‐benzoquinone imine.

All patients with harmful alcohol intake should have a clearly documented plan for safe discharge following day surgery. Consideration should be given to social circumstances in high‐risk patients, given the potential for home alcohol withdrawal and the interaction of alcohol consumed at home with peri‐operative medications. Such patients may not be suitable for ‘home alone’ discharge as described elsewhere and should return home with a responsible adult [35]. Nurse‐led discharge on the day of surgery should otherwise follow explicit criteria according to local protocols, with additional vigilance for signs of withdrawal in the recovery area. Provision of written patient discharge material, with details of surgical procedure, follow‐up planning and key contacts such as community alcohol liaison services as relevant, is critical for ongoing patient safety.

#### Emergency surgery

It may not be practical or safe to implement some of the pre‐operative measures described above, such as controlled alcohol withdrawal, in patients who require time‐critical or emergency surgery. However, it should be noted that acute presentation does not preclude judicious pre‐operative intervention, depending on clinical context. Furthermore, the identification of patients with excessive alcohol intake also influences acute management within the operating theatre and after surgery (Fig. 3). If feasible, screening patients rapidly with the AUDIT‐C questionnaire during anaesthetic assessment is therefore important for prompt recognition, early involvement of other key specialities and tailoring of subsequent therapy.

### Optimisation

#### General considerations

Consent should include details of the additional risks of alcohol withdrawal and postoperative complications. Patients who are intoxicated, encephalopathic or have chronic alcohol‐related brain damage may not have sufficient mental capacity to provide consent specific to the intended procedure. Management should incorporate the patient's best interests and, if appropriate, an attempt at substituted judgement regarding their wishes. These elements should align with the principles of the Mental Capacity Act (2005) in keeping with existing guidance published by the Association of Anaesthetists [36].

Patients with cirrhosis should be discussed with gastroenterology or hepatology specialists where possible. The severity of cirrhosis correlates with the risk of postoperative mortality [37] and a careful history should be taken for features of hepatic decompensation. The risk calculators discussed previously may assist with planning for these cases. However, such tools should be treated as adjuncts to clinical decision‐making; it may be most appropriate to refer complex and/or decompensated patients to a liver centre where there is immediately available subspecialty expertise. In the elective setting, appropriately timed optimisation for this cohort may include treatment of ascites; screening and prophylaxis of varices; treatment of hepatic encephalopathy; and nutritional intervention.

Balanced correction of nutritional, metabolic, fluid and haematological abnormalities will require multidisciplinary input, with referral to relevant specialist teams for organ‐specific complications. The detailed peri‐operative management of deranged physiology in liver disease is covered in a recent publication [19]. Issues most relevant to patients with harmful alcohol intake are discussed below.

#### Nutritional issues

The direct effects of harmful alcohol intake may compound with secondary liver disease to profoundly disrupt energy and nutritional homeostasis. Resultant malnutrition is common, occurring in 60–85% of patients with cirrhosis and encompassing a spectrum of conditions including sarcopenia; frailty; electrolyte abnormalities; and vitamin deficiencies. Underlying mechanisms are multiple and interdependent, including appetite loss; intestinal mucosal/microbiome disruption with resultant malabsorption; and hypercatabolic states secondary to oxidative alcohol metabolism and systemic, low grade bacterial translocation [38, 39].

Nutritional status should therefore be considered in all patients with harmful alcohol intake. There is no gold standard tool for screening this population for malnutrition. The Malnutrition Universal Screening Tool is recommended by the European Society for Clinical Nutrition and Metabolism, although BMI calculations may be confounded by the presence of ascites. Alternative screening tools adapted for liver disease, such as the Royal Free Hospital Nutritional Prioritisation Tool, may present a useful alternative if there is diagnostic uncertainty [39].

Although detailed discussion regarding management is beyond the scope of this document, general considerations include energy requirements; protein intake; electrolyte replacement; vitamin supplementation; and micronutrients. Associated hepatic complications, such as alcoholic hepatitis, ascites or encephalopathy may alter individual needs in these domains [39]. Harmful alcohol intake is also an independent risk factor for the development of refeeding syndrome [40], marked by potentially life‐threatening fluid and electrolyte shifts precipitated by over‐supplementation in susceptible individuals. Appropriate involvement of a dietician for complex cases is advised throughout the peri‐operative period. It should also be noted that infusions of ferric carboxymaltose (Ferinject®, Vifor Pharma UK Ltd, Staines, UK) for iron replacement may present a particular risk of hypophosphataemia for some patients in this group due to pre‐existing phosphate depletion/concurrent refeeding syndrome.

Regular thiamine‐containing supplements, such as Pabrinex® (Kyowa Kirin Ltd, Galashiels, UK), should be prescribed at admission to patients admitted with chronic harmful alcohol consumption to prevent Wernicke's encephalopathy, a serious complication of alcohol misuse comprising ophthalmoplegia, ataxia and acute delirium precipitated by vitamin B deficiency. Regimens may vary by institution but would typically include intravenous therapy for 72 h followed by conversion to oral supplementation (Fig. 3).

#### Haematological issues

The treatment of coagulation abnormalities in patients with cirrhosis before surgery has become increasingly complex. For low‐risk procedures, routine administration of blood products such as fresh frozen plasma to achieve a specific laboratory coagulation value has little benefit and may cause harm. Where bleeding is considered likely, such as in those undergoing major surgery, it is reasonable to target specific endpoints such as a platelet count of > 50 × 10^9^ cells.l^‐1^ or a fibrinogen concentration of > 1 g.l^‐1^ in decompensated disease. Appropriate haematological involvement, supplemented by investigations such as viscoelastic testing, is critical for decision‐making in complex cases [19, 41].

The literature on the efficacy and safety of peri‐operative tranexamic acid in patients with chronic harmful alcohol intake and associated liver disease is incomplete. Recent recommendations from NICE advise routine prophylaxis in particular circumstances, in keeping with several large meta‐analyses [42]. This contrasts with a recent, large multicentre randomised controlled trial indicating that tranexamic acid use in acute upper gastrointestinal haemorrhage, a known complication of cirrhosis, does not reduce 5‐day mortality from bleeding and may slightly increase the risk of venous thromboembolism and seizures [43]. The authors highlighted the divergent findings in this study compared with a preceding Cochrane review, attributing this in part to the potential for erroneous results in meta‐analyses of small studies [44].

Literature examining tranexamic acid use for the management of bleeding in other emergency settings predominantly consists of single‐centre studies and has not demonstrated an increased thrombotic risk, although high doses may increase the risk of seizures [45]. These studies do not specifically focus on patients with harmful alcohol intake or liver disease and the true risk/benefit of antifibrinolytic therapy in this group therefore remains controversial. In the absence of definitive evidence or a specific contraindication, it remains reasonable to give tranexamic acid for the prevention or management of acute haemorrhage in most circumstances. Avoidance of high doses is advised where seizure risk is elevated, such as acute withdrawal states or when patients are at risk of this.

### Peri‐operative management

#### Intra‐operative

The systemic effects of harmful alcohol intake, in combination with other potential issues such as acute illness and secondary organ dysfunction, may warrant modification of anaesthesia techniques (Table 2). It should be noted that there are few outcome data supporting neuraxial techniques over general anaesthesia in patients with established liver disease [19].

**Table 2:** Anaesthetic considerations for patients with harmful alcohol intake (with or without associated organ disease) particularly those with hepatic, cardiac and autonomic dysfunction.

	Issue	Action
Airway management	Risk factors for aspiration include acute intoxication and chronic autonomic neuropathy with delayed gastric emptying	Perform rapid sequence induction for patients who are acutely intoxicated and undergoing general anaesthesia. Aspiration risk should also be considered when forming an extubation strategy
		Evidence for rapid sequence induction in patients with chronic harmful intake and who are stable is less clear. Advisable to intubate the trachea of all patients in this group and consider rapid sequence induction if there is a history of symptomatic reflux
		Consider pre‐treatment with anti‐reflux prophylaxis
Cardiovascular	Higher risk for haemodynamic instability due to autonomic neuropathy; cardiomyopathy; pulmonary hypertension; reduced systemic vascular resistance (in severe alcohol‐related liver disease); and dehydration in acute intoxication	Site large bore intravenous access pre‐induction with provision for rapid fluid administration if needed
		Have a lower threshold for invasive intra‐arterial monitoring in addition to standard monitoring
		If large fluids shifts are anticipated, consider cardiac output monitoring
		Consider pre‐emptive vasopressor infusions to mitigate hypotension
	Increased bleeding risk from hepatic coagulopathy; platelet dysfunction; and portal hypertension	If appropriate, ensure rapid availability of cross‐matched blood and other blood products as required such as vitamin K, clotting factors, fresh frozen plasma and platelets
		Judicious use of tranexamic acid to treat or prevent major haemorrhage. Balanced decision‐making for administration and dosage in alcohol withdrawal
Neurological	Patients may have significant pre‐operative anxiety that can indicate acute withdrawal	Consider an anxiolytic such as midazolam to treat potential withdrawal and provide a dose‐sparing effect for other induction drugs when performing general anaesthesia. Use reduced doses of these drugs in advanced liver disease
	Patients with chronic harmful alcohol intake under general anaesthesia are still at risk of withdrawal intra‐procedurally	Vigilance for signs of alcohol withdrawal or seizures during maintenance of general anaesthesia. Signs include tachypnoea; hypercarbia; tachycardia; hypertension; hyperpyrexia; and sweating
	Established liver disease and/or malnourishment increases the risk of intra‐operative hypoglycaemia	Hourly glucose monitoring is advisable

Given the challenges encountered in patients with harmful alcohol consumption, it may be more appropriate to consider performing surgery using regional or local anaesthetic techniques. Potential intra‐ and postoperative benefits include avoidance of the cardiorespiratory and neurological effects of general anaesthesia in a high‐risk cohort; enhanced seizure detection in patients who are awake; and mitigation of pharmacological effects of systemic medications. Due consideration should be given to factors influencing the suitability of regional techniques in these patients, including appropriateness of techniques for surgical procedures and their duration; reduced patient cooperation or reduction in mental capacity due to intoxication or chronic neurological impairment; presence of coagulopathy and/or thrombocytopenia; and the influence of hypoproteinaemia (secondary to malnutrition and/or hepatic synthetic dysfunction) on local anaesthetic distribution and dosing limits.

#### Pharmacological issues

Chronic harmful alcohol intake and related organ dysfunction, as well as acute intoxication, may significantly influence the efficacy and safety of commonly used medications, with a commensurate requirement to modify or avoid their use. Where there is alcohol‐related secondary organ dysfunction, judicious administration of intra‐operative anaesthetic drugs is warranted (Table 3). This reflects vulnerability to the cardiorespiratory effects of such medications, as well as the widely recognised changes in drug pharmacokinetics seen in alcohol‐related liver disease.

**Table 3:** Impact of harmful alcohol intake and secondary organ dysfunction on anaesthetic drugs used intra‐operatively.

	Potential issues
Propofol	Reduced dosing in acute intoxication, cardiac and hepatic failure Increased dosing requirements in chronic excessive alcohol intake
Thiopentone	Reduced dosing in acute intoxication, cardiac and hepatic failure No evidence of altered doses in chronic excessive alcohol intake
Etomidate	No evidence of altered doses in chronic excessive alcohol intake
Inhalational anaesthetics	Neurological depression due to intoxication decreases minimum alveolar concentration requirements
Neuromuscular blocking drugs	Altered volume of distribution and protein binding in severe chronic liver disease may result in increased dosing requirements Atracurium is generally safest due to non‐hepatic metabolism and excretion Vecuronium and rocuronium are hepatically metabolised and should be used with caution Suxamethonium may show increased duration of action in severe alcohol‐related liver disease due to plasma cholinesterase deficiency

#### Postoperative

Unhealthy alcohol intake contributes significantly to postoperative complications, in particular alcohol withdrawal syndrome, postoperative infections and delirium [46, 47]. This leads to an increased duration of hospital stay, ICU admissions and mortality [46, 47]. This picture is exacerbated by the presence of related comorbidities, decompensation of which may precipitate hepatic encephalopathy, cardiac ischaemia and arrhythmias [47].

Patients should therefore be monitored to detect complications, with a low threshold for postoperative admission to critical care. The decision to refer for critical care support should be guided using a peri‐operative risk calculator, such as P‐POSSUM, which should be appropriately repeated at the end of surgery to account for determinant intra‐procedural events such as major haemorrhage. It should be noted that liver‐specific scoring systems outlined earlier have primary utility in pre‐operative discussion and referral; they are not suitable for dynamic assessment of risk and determination of postoperative location for patients undergoing procedures.

The incidence of alcohol withdrawal syndrome is estimated to be 2–5 times higher in surgical patients compared with other inpatients and has a higher associated morbidity [47, 48]. Features range from minor symptoms such as headache, tremor, and insomnia, to more serious manifestations including withdrawal seizures, hallucinations and delirium tremens. Approximately 5% of patients who undergo alcohol withdrawal suffer from delirium tremens and mortality is significant if left untreated [33]. At‐risk patients should be monitored using the described CIWA‐Ar scale or equivalent, with ongoing consideration for thiamine supplementation [27, 33]. Where available, continued oversight from alcohol support services is advised.

Patients with established alcohol‐related liver disease are at increased risk of postoperative morbidity and mortality, a picture complicated by the potential impact of both hepatic dysfunction and continued harmful alcohol intake on postoperative analgesia (Table 1) [33, 34]. Hepatic encephalopathy can be precipitated by hypoxia, hypovolaemia or acid–base/electrolyte disturbance, which should be avoided during and after surgery [49]. Infections may also provoke hepatic decompensation, warranting vigilance and aggressive treatment when present. Patients with cirrhosis are also at high risk of renal dysfunction, necessitating caution when using nephrotoxic agents and judicious fluid balance, aiming to preserve renal perfusion while avoiding volume overload.

## Conclusions

Patients with harmful alcohol intake represent a complex and challenging population, mandating a multidisciplinary team‐based approach at all stages in a cohort whose disease may be clinically silent until unmasked by surgical stressors. Safety depends on an understanding of the direct and indirect effects of alcohol intake on physiology and pharmacology, with the use of validated tools to identify those who would benefit from withdrawal management and targeted critical care involvement in the immediate peri‐operative phase.
